# Growing evidence of pharmacotherapy effectiveness in managing attention-deficit/hyperactivity disorder in young children with or without autism spectrum disorder: a minireview

**DOI:** 10.3389/fpsyt.2024.1408876

**Published:** 2024-06-24

**Authors:** Hamza A. Alsayouf

**Affiliations:** Dr. Hamza Alsayouf Clinic, Amman, Jordan

**Keywords:** alpha-2 adrenergic agonists, aripiprazole, atomoxetine, attention-deficit/hyperactivity disorder, autism spectrum disorder, methylphenidate, pharmacotherapy, risperidone

## Abstract

Many children with autism spectrum disorder (ASD) also have attention-deficit/hyperactivity disorder (ADHD). ADHD in children is associated with increased risk of negative outcomes, and early intervention is critical. Current guidelines recommend psychosocial interventions such as behavioral training as the first line of therapy in managing ADHD symptoms in children with or without ASD. Where symptoms are refractory to these interventions, medications such as stimulants, α2-adrenergic agonist inhibitors, selective norepinephrine reuptake inhibitors, and second-generation antipsychotics are recommended. However, these pharmacotherapies do not have regulatory approval for use in children of preschool age, and evidence on their safety and efficacy in this population has historically been very limited. Since publication of the current guidelines in 2020, several new randomized controlled trials and real-world studies have been published that have investigated the efficacy and tolerability of these medications in preschool children with ADHD, with or without comorbid ASD. Here, we provide a review of the key findings of these studies, which suggest that there is growing evidence to support the use of pharmacological interventions in the management of ADHD in preschool children with comorbid ASD.

## 1 Introduction

Attention-deficit/hyperactivity disorder (ADHD) is the most commonly diagnosed neurodevelopmental condition in childhood (1) and occurs in 40–70% of children with autism spectrum disorder (ASD) (2). There is a marked overlap in symptoms between ADHD and ASD (3) that is suggestive of a related etiology (4–6), with similar alterations observed in e.g., neural connectivity, sensory processing, sleep patterns, and motor and impulse control.

Updates to the 5th edition of the Diagnostic and Statistical Manual of Mental Disorders (DSM-5) criteria now allow the diagnosis of ADHD in children with a comorbid diagnosis of ASD (7, 8). It is hoped that this will allow more children to be fully diagnosed and treated effectively (1, 8, 9). Standard ADHD rating scales have not yet been validated for patients with comorbid ASD, and careful differential diagnosis of ADHD is required based on observation of the child in a variety of settings (9).

ADHD is characterized by hyperactivity, inattention, and impulsivity (7), which can have lasting negative effects that extend into adulthood (10, 11). Preschool children with ADHD tend to have difficulties in developing fine motor abilities and foundational skills (e.g., writing, basic mathematics, and prereading), and they struggle to socialize due to aggression, disruptiveness, and impulsivity (10, 12). Symptoms of ADHD can exacerbate and perpetuate impairments in children with comorbid ASD, further increasing the risk of negative outcomes in these children and placing an even greater burden on families and caregivers (9).

Several medications have been evaluated in clinical trials for efficacy in the management of ADHD symptoms. Many of these may also have a beneficial effect in patients with comorbid ASD and mainly include stimulants, selective norepinephrine reuptake inhibitors, α-2 adrenergic agonists, and second-generation antipsychotics (13). However, the only pharmacological therapies that the United States’ Food and Drug Administration (FDA) have approved are risperidone (approved 2006) and aripiprazole (approved 2009) for the treatment of children with ASD from the ages of 5 and 6 years, respectively, and these medications are indicated for the comorbid symptoms of irritability and aggression that are frequently associated with this condition (14, 15).

Currently, the most recent guidelines from the American Academy of Pediatrics (AAP) from 2020 recommend that psychosocial interventions (e.g., parent training and behavioral classroom interventions) be used as first-line therapy in children with ASD to manage their core symptoms and any comorbid challenging behaviors, with pharmacotherapies to be used as an adjunct where such interventions are not sufficiently effective (1, 9). However, due to the many potential barriers in place related to access to non-pharmacological treatment strategies (16), combined with the increasing number of children diagnosed with ASD (17), most clinicians do not follow these guidelines (16), and many preschool children either receive medications off-label or remain untreated (18). Nevertheless, there is consensus that early diagnosis and treatment are associated with better social, mental health, educational, and functional outcomes in children with ADHD and ASD (19–21).

The purpose of this mini-review is to describe recently published evidence (2020 to present day) of the efficacy and safety of pharmacotherapies in the treatment of ADHD symptoms in young children ≤6 years. Data from both randomized controlled trials (RCTs) and key observational studies will be discussed, with a particular focus on those including children with comorbid ASD. Given the increasing prevalence of ASD and its associated comorbidities such as ADHD (17), there is a growing need for therapeutic strategies that are both effective and safe in this population.

## 2 Recent evidence on ADHD medications

### 2.1 Stimulants

Methylphenidate and amphetamine-based stimulants are among the most commonly prescribed medications for ADHD in children and several formulations are approved for the management of ADHD symptoms (22). For children with ASD, the AAP 2020 guidelines recommend stimulants for the treatment of ADHD symptoms, particularly hyperactivity and impulsivity, where previous, non-pharmacological interventions have not been effective (9).

Until recently, the PATS study (23) was the main reference study on the use of stimulants in preschool children with ADHD. However, there has been an upsurge in interest in stimulants, with several RCTs being published since 2020 in this population (24–26). In addition, the first systematic literature review and meta-analysis to evaluate stimulants in preschool children was published in 2023 (27). It identified five RCTs (including the three published since 2020) across several literature databases and clinical trial registries from inception to 2022 (total n=489; mean age, 4.95 years) that compared stimulants to placebo in preschool children ≤7 years of age in the treatment of ADHD. These double-blinded RCTs assessed three methylphenidate immediate‐release, one methylphenidate extended‐release, and one lisdexamfetamine formulation. The analysis found that stimulants provided a significant benefit over placebo in terms of reduction in ADHD symptom severity (standardized mean difference = −0.59; 95% CI −0.77, −0.41; p < 0.0001), and the evidence was considered of moderate robustness. The authors concluded that although the effect size was smaller than that reported in school-age children and adolescents (28), stimulants are efficacious in reducing ADHD symptoms among preschool children. The meta-analysis by Cortese et al. in older children suggested methylphenidate as the first line of pharmacological therapy for ADHD (28), but in the meta-analysis of preschool children by Sugaya et al., the analyses were underpowered to detect significant differences in the effect of methylphenidate and amphetamines in this population (27).

In addition, the meta-analysis by Sugaya et al. found that the use of stimulants did not lead to an increased rate of all‐cause discontinuations of treatment in comparison with placebo, but confidence in this estimate was very low (27). Although the study could not robustly analyze stimulant tolerability, results from the individual RCTs indicated that short-term treatment with stimulants was generally well tolerated (the median treatment duration was 4 weeks), with most adverse events considered to be mild or moderate.

Reported adverse events that would require close monitoring to address concerns regarding long-term effects, are increased heart rate and blood pressure and decreased growth rates: in the MAPPA study (26), an increase in blood pressure and heart rate was reported in 2 (3.9%) and 1 (2.0%) of the patients, respectively, who were treated with methylphenidate plus sham behavioral parental training for 8 weeks; however, the rates of these cardiovascular events were not significantly different from those in the placebo arms. In the PATS study (n=183) (29), although blood pressure was observed to increase over 1 year of treatment, there were no cases that met the predefined criteria for clinically significant hypertension, and there were no significant differences observed in the rates of elevated blood pressure readings between the treatment and placebo groups. The study by Childress et al. (24) had no placebo arm; 6 (6.7%) patients experienced hypertension, 3 (3.4%) had prehypertension, and 2 (2.2%) had tachycardia; one case (1.1%) of tachycardia was not considered serious by the study investigator but led to treatment discontinuation. Childress et al. also observed a decline in weight and height over 12 months of treatment, with weight loss reported as an adverse event in 16 (18.0%) children and leading to treatment discontinuation in 2 (2.2%) children. The MAPPA study (26) similarly observed a decline in weight and body mass index from baseline in the treatment arm, but there were no significant differences in these measures between the treatment and placebo groups at the study endpoint. In the PATS study (29), weight loss as an adverse event was reported significantly more frequently in children in the treatment arm vs the placebo arm, and 1 child discontinued treatment due to weight loss.

A large retrospective study based on claims data from 2000–2016 in the United States in children aged 3–18 years with ADHD or ASD found no evidence of increased serious cardiovascular risk associated with exposure to stimulants or atomoxetine (30). Importantly however, the long-term effects of medication may differ when initiated at different stages of development (31), and further prospective studies are required to determine whether children exposed to stimulants at an early age develop persistent cardiovascular complications.

There is no recent evidence from RCTs on the use of stimulants in the treatment of ADHD comorbid with ASD in preschool children. A retrospective chart review by Harstad et al. (32) compared α2-adrenergic agonists and stimulants in preschool children with or without ASD, in which both classes of medication were found to be effective in improving ADHD symptoms. For stimulants, the most commonly reported adverse events in young children were increased moodiness/irritability and appetite suppression, which was consistent with findings from RCTs in other age groups.

Overall, stimulants appear to be effective and well tolerated in preschool children and should be considered for the management of ADHD symptoms in children with ASD. However, the side effect profile of stimulants can be worse in children with ASD, and stimulants may appear less effective as a result of exacerbation of coexisting symptoms such as social anxiety, insomnia, reduced appetite, and irritability (9, 24, 32–35). A complete medical evaluation is required along with a comprehensive assessment of the child’s personal and family history of cardiac disease, and the child’s weight, height, blood pressure, and heart rate should be measured at each follow-up visit (27). Routine cardiology consultations or assessments such as electrocardiograms should not be necessary unless indicated in individual cases (36). Treatment should be initiated with small doses that are carefully and slowly titrated up if needed to achieve sufficient symptom control, and care must be taken to avoid unnecessarily large doses (9, 22).

### 2.2 α2-Adrenergic agonists

Despite the frequency at which α2-adrenergic agonists such as clonidine and guanfacine are prescribed to preschool children with ADHD, either alone or in combination with stimulants (22), there have been no blinded RCTs to date on their use in this population. Observational studies such as the recent retrospective chart review by Harstadt et al. (32) support the effectiveness of α2-adrenergic agonists in young children with ADHD, with or without comorbid ASD, and the most commonly reported adverse events in this study were daytime sleepiness and increased moodiness/irritability. When combined with stimulants, daytime sleepiness can be minimized. However, there remains a paucity of evidence on long-term efficacy and safety in preschool children. Data from studies in older children indicate that treatment with α2-adrenergic agonists requires a comprehensive evaluation and family history, along with close monitoring for cardiovascular adverse events such as hypotension, bradycardia, and QTc prolongation (36).

The AAP 2020 guidelines for ASD recommend the use of α2-adrenergic agonists in children with ASD for targeting ADHD symptoms such as hyperactivity and impulsivity, where these symptoms do not respond to treatment with stimulants or where adverse effects are a concern (9). However, retrospective chart reviews indicate that preschool children with ADHD and comorbid ASD are more likely to be prescribed α2-adrenergic agonists than stimulants, whereas stimulants are more likely to be prescribed to those without comorbid ASD (37, 38). This may reflect clinicians’ and caregivers’ concerns regarding stimulants possibly affecting children’s sleep, as sleep disorders are common in children with ASD (39). Furthermore, α2-adrenergic agonists are also beneficial in managing stereotypical behavior and tics, insomnia, aggression, depression, and anxiety, which makes these medications favorable for use in children with ASD and comorbid ADHD (9, 40–43).

### 2.3 Selective norepinephrine reuptake inhibitors

Atomoxetine is a selective norepinephrine reuptake inhibitor (SNRI) that has been shown to be effective in the treatment of ADHD in school-age children, adolescents, and adults (44), and it is currently approved for the treatment of ADHD in children aged 6 years and older (45). To date, however, there has been only one double‐blind RCT that has compared atomoxetine and placebo for ADHD treatment in preschool children (46). In this study, significant improvements in ADHD symptoms were reported following 8 weeks of treatment in 101 children of 5 to 6 years of age, but response to treatment was highly variable, with many children remaining moderately to severely impaired by the end of the study. However, longer-term treatment may be needed to fully assess atomoxetine efficacy (47). More recently, in an observational study of atomoxetine in 133 children aged 3–6 years with ADHD comorbid with ASD, atomoxetine titrated to an individualized dose of 1.2–1.8 mg/kg/day appeared to be effective, with an improvement in ADHD symptoms observed in most patients (n=110) who completed at least 6 months of treatment (48).

In the RCT published by Kratochvil et al. (46), the most commonly reported adverse events in the atomoxetine group were gastrointestinal upset and decreased appetite. These adverse effects were mostly mild to moderate in severity. In the observational study by Alsayouf et al. (48), the most common adverse events were gastrointestinal, aggression or hostility, and increased hyperactivity; in all patients who discontinued treatment due to adverse events, symptoms related to these events resolved after treatment ceased. These safety findings are consistent with those from an earlier open-label study (49).

An increased risk of suicidal ideation was observed in children in a pooled analysis of the key clinical trials by Eli Lilly (0.4% vs 0% for atomoxetine vs placebo), with one attempted suicide reported (50). These events occurred during the first month of atomoxetine treatment. As a result, the product label includes a warning that children should be monitored carefully for behavioral changes, particularly when initiating treatment or altering the dose (increasing or decreasing) (45). A subsequent observational study of 4509 children in the United Kingdom (median age, 11 years) found no evidence of an increased risk of suicidal ideation associated with atomoxetine treatment (51).

Adverse events during atomoxetine treatment tend to occur more frequently in preschool children than in older children, and this may be due to developmental differences in pharmacokinetics (52). Furthermore, there is broad heterogeneity in response to atomoxetine in terms of efficacy and tolerability, and this is likely attributable in large part to atomoxetine being mainly metabolized by the highly polymorphic enzyme, Cytochrome P450 2D6 (52, 53). Ultra-rapid metabolizers may experience reduced efficacy, and poor metabolizers may have a higher risk of adverse events. Therefore, it is important to slowly titrate the medication to find the optimal dose for each child.

Thus, atomoxetine may be considered for the treatment of ADHD symptoms in preschool children where symptoms remain refractory to stimulants as a first-line therapy, and it is particularly recommended in children where social anxiety and obesity are also present (9, 54). The initial dose should be low and slowly titrated up, and children should be monitored for adverse effects, including any changes in behavior, particularly when the dose is changed.

### 2.4 Second-generation antipsychotics

Risperidone and aripiprazole are currently the only medications approved by the FDA for the treatment of the comorbid symptoms of irritability and aggression in children with ASD, but only from the ages of 5 and 6 years, respectively (13–15). These second-generation antipsychotic medications are also recommended in the AAP 2020 guidelines for the treatment of hyperactivity in children with ASD, for patients in whom stimulants, atomoxetine, and α2-adrenergic agonists as first-line and second-line therapies are ineffective or not well tolerated (9).

Although there have been many clinical trials on the use of risperidone and aripiprazole for the treatment of various challenging behaviors in children with ASD aged 5–18 years (13, 55), there is very little evidence from RCTs in preschool children, including in the management of comorbid ADHD symptoms in this population (27, 56). Despite the lack of RCT-based evidence and regulatory approval, antipsychotic medications are increasingly prescribed to preschool children with ASD, with HCPs relying on reported clinical experience instead (57–61). Two recent observational studies on treatment outcomes with antipsychotics in children with ASD (mean age <6 years) suggest that risperidone and aripiprazole are effective in treating both the core signs and symptoms of ASD, as well as comorbid challenging behaviors such as hyperactivity and inattention; adjunct treatment with methylphenidate or atomoxetine was also prescribed as needed to help manage weight gain and improve attention (62, 63). Notably, recent RCT evidence from school-age children suggests that optimizing the dose of any stimulant prescribed prior to initiating antipsychotics is fundamental for achieving beneficial outcomes (64). Antipsychotic medications can be particularly useful in patients with comorbid symptoms such as anxiety, irritability, depression, aggression, self-harm, repetitive behaviors, and behavioral rigidity (9).

It is important to note that treatment with antipsychotics may result in potentially long-term adverse effects that include weight gain, hyperprolactinemia, cardiac symptoms such as tachycardia or a prolonged QTc interval, extrapyramidal symptoms such as dystonia or tardive dyskinesia, and metabolic disturbances such as hyperlipidemia or hyperglycemia (13, 65, 66). Thus, families and clinicians need to carefully consider the potential benefits and risks of using these medications when young children are unresponsive to other treatment strategies. A comprehensive medical examination, thorough history, as well as close monitoring with regular follow-up are required in children undergoing antipsychotic treatment. Before starting treatment, it is recommended to perform the relevant laboratory tests to generate a baseline against which any metabolic adverse effects can be monitored during subsequent checkups (67–71), and an echocardiogram and electrocardiogram should be performed on all patients when the maximum dose is reached or earlier if indicated (72–75). At every visit, weight, body mass index, height, and vital signs should be assessed, and an evaluation such as the Abnormal Involuntary Movement Scale (AIMS) should be conducted (76).

## 3 Conclusions

Children with ADHD face significant impairment and an increased risk of mortality, morbidity, and poor educational outcomes (10, 11, 77). The early diagnosis and treatment of ADHD in preschool children has the potential to markedly improve their long-term quality of life by preventing a downward spiral of increasing impairment and comorbidity (11).

Current guidelines recommend psychosocial interventions as the first line of therapy for children with ASD, with pharmacological treatment only recommended as needed when previous interventions are not sufficiently effective (9). However, psychosocial interventions are not widely available or may be cost-prohibitive, particularly in resource-limited regions, and the early evidence base for these is arguably outdated and/or lacking in robustness (27). Treating ADHD in children with ASD is as important as treating ADHD in children without ASD, and the same medications can be used for both groups of patients (9, 78). Since these guidelines have been published, there have been several RCTs and real-world studies examining pharmacological interventions for ADHD in children of preschool age, particularly for stimulants.

Regardless of the treatment chosen, it is important to emphasize that there remains limited evidence on the long‐term efficacy and safety of ADHD medications in preschool children. In addition, children with comorbid ASD appear to be more susceptible to adverse effects from ADHD medications than those with ADHD alone (9). For this reason, medications should be slowly titrated up from a small initial dose, and children need to be carefully monitored by their families, caregivers, and HCPs for adverse effects. Adverse event monitoring by clinicians should include regular measures of height, weight, heart, and blood pressure; where indicated, further cardiologic assessments may be required. For treatment with antipsychotics, metabolic indicators should also be monitored. Clinicians need to also provide regular follow-ups to assess any changes in ADHD/ASD symptom severity and global functioning.

Pharmacological intervention for the treatment of ADHD symptoms comorbid with ASD in very young children requires transparent communication and collaborative decision-making between families and HCPs (9). It is critical that treatment strategies be individualized, considering not only the available evidence on efficacy and safety, but also each child’s symptom severity, comorbidities, and functional needs. Other factors that will play a role include treatment cost and availability, parenting practices, and family preferences. Families also need to be fully informed of the potential long-term negative consequences of not treating/delaying treatment of ADHD symptoms. Future, long-term RCTs in preschool children are necessary to provide more rigorous, evidence-based recommendations for this population.

## Author contributions

HA: Writing – original draft, Writing – review & editing.

## References

[1] Wolraich ML Hagan JF Jr. Allan C Chan E Davison D Earls M. Subcommittee on Children and Adolescents with Attention-Deficit/Hyperactive Disorder. Clinical practice guideline for the diagnosis, evaluation, and treatment of attention-deficit/hyperactivity disorder in children and adolescents. Pediatrics. (2019) 144:e20192528. doi: 10.1542/peds.2019-2528 31570648 PMC7067282

[2] Schachar RJ Dupuis A Arnold PD Anagnostou E Kelley E Georgiades S. Autism spectrum disorder and attention-deficit/hyperactivity disorder: Shared or unique neurocognitive profiles? Res Child Adolesc. Psychopathol. (2023) 51:17–31. doi: 10.1007/s10802-022-00958-6 36006496 PMC9763138

[3] Miodovnik A Harstad E Sideridis G Huntington N. Timing of the diagnosis of attention-deficit/hyperactivity disorder and autism spectrum disorder. Pediatrics. (2015) 136:e830–7. doi: 10.1542/peds.2015-1502 26371198

[4] Kern JK Geier DA Sykes LK Geier MR Deth RC. Are ASD and ADHD a continuum? A comparison of pathophysiological similarities between the disorders. J Atten. Disord. (2015) 19:805–27. doi: 10.1177/1087054712459886 23074304

[5] Kern JK Geier DA King PG Sykes LK Mehta JA Geier MR. Shared brain connectivity issues, symptoms, and comorbidities in autism spectrum disorder, attention deficit/hyperactivity disorder, and Tourette syndrome. Brain Connect. (2015) 5:321–35. doi: 10.1089/brain.2014.0324 25602622

[6] Hoogman M Van Rooij D Klein M Boedhoe P Ilioska I Li T. Consortium neuroscience of attention deficit/hyperactivity disorder and autism spectrum disorder: The ENIGMA adventure. Hum Brain Mapp. (2022) 43:37–55. doi: 10.1002/hbm.25029 32420680 PMC8675410

[7] American Psychiatric Association. Diagnostic and Statistical Manual of Mental Disorders: DSM-5. 5th ed. Washington, DC, USA: American Psychiatric Association (2013).

[8] Eom TH Kim YH. Clinical practice guidelines for attention-deficit/hyperactivity disorder: Recent updates. Clin Exp Pediatr. (2024) 67:26–34. doi: 10.3345/cep.2021.01466 37321571 PMC10764666

[9] Hyman SL Levy SE Myers SM. Council on Children with Disabilities, Section on Developmental and Behavioral Pediatrics. Identification, evaluation, and management of children with autism spectrum disorder. Pediatrics. (2020) 145:e20193447. doi: 10.1542/9781610024716 31843864

[10] Varrasi S Boccaccio FM Guerrera CS Platania GA Pirrone C Castellano S. Schooling and occupational outcomes in adults with ADHD: Predictors of success and support strategies for effective learning. Educ Sci. (2023) 13:37. doi: 10.3390/educsci13010037

[11] Sonuga-Barke EJ Koerting J Smith E McCann DC Thompson M. Early detection and intervention for attention-deficit/hyperactivity disorder. Expert Rev Neurother. (2011) 11:557–63. doi: 10.1586/ern.11.39 21469928

[12] Egger HL Angold A. Common emotional and behavioral disorders in preschool children: Presentation, nosology, and epidemiology. J Child Psychol Psychiatry Allied Discipl. (2006) 47:313–37. doi: 10.1111/j.1469-7610.2006.01618.x 16492262

[13] Anagnostou E. Clinical trials in autism spectrum disorder: evidence, challenges and future directions. Curr Opin Neurol. (2018) 31:119–25. doi: 10.1097/WCO.0000000000000542 29389748

[14] Food and Drug Administration. Approval for Risperdal (risperidone) in treatment of the irritability associated with autistic disorder. Available online at: https://www.accessdata.fda.gov/drugsatfda_docs/nda/2006/020272Orig1s036,s041,020588Orig1s024,s028,s029,21444Orig1s008,s015.pdf (Accessed 19 March 2024).

[15] Bristol Myers Squibb. U.S. Food and Drug Administration approves ABILIFY® (aripiprazole) for the treatment of irritability associated with autistic disorder in pediatric patients (ages 6 to 17 Years). Available online at: https://news.bms.com/news/details/2009/US-Food-and-Drug-Administration-Approves-ABILIFY-aripiprazole-for-the-Treatment-of-Irritability-Associated-with-Autistic-Disorder-in-Pediatric-Patients-Ages-6-to-17-Years/default.aspx (Accessed 19 March 2024).

[16] Moran A Serban N Danielson ML Grosse SD Cuffe SP. Adherence to recommended care guidelines in the treatment of preschool-age Medicaid-enrolled children with a diagnosis of ADHD. Psychiatr Serv. (2019) 70:26–34. doi: 10.1176/appi.ps.201800204 30373494 PMC6408287

[17] GBD 2019 Mental Disorders Collaborators. Global, regional, and national burden of 12 mental disorders in 204 countries and territories, 1990–2019: A systematic analysis for the Global Burden of Disease Study 2019. Lancet Psychiatry. (2022) 9:137–50. doi: 10.1016/S2215-0366(21)00395-3 PMC8776563 35026139

[18] Danielson ML Visser SN Gleason MM Peacock G Claussen AH Blumberg SJ. A national profile of attention-deficit hyperactivity disorder diagnosis and treatment among US children aged 2 to 5 years. J Dev Behav Pediatr. (2017) 38:455–64. doi: 10.1097/DBP.0000000000000477 PMC5598086 28723824

[19] Boland H DiSalvo M Fried R Woodworth KY Wilens T Faraone SV. A literature review and meta-analysis on the effects of ADHD medications on functional outcomes. J Psychiatr Res. (2020) 123:21–30. doi: 10.1016/j.jpsychires.2020.01.006 32014701

[20] Bradshaw J Steiner AM Gengoux G Koegel LK. Feasibility and effectiveness of very early intervention for infants at-risk for autism spectrum disorder: a systematic review. J Autism Dev Disord. (2015) 45:778–94. doi: 10.1007/s10803-014-2235-2 25218848

[21] Knott R Mellahn OJ Tiego J Kallady K Brown LE Coghill D. Age at diagnosis and diagnostic delay across attention-deficit hyperactivity and autism spectrums. Aust N Z. J Psychiatry. (2024) 58:142–51. doi: 10.1177/00048674231206997 PMC10838471 37885260

[22] Panther SG Knotts AM Odom-Maryon T Daratha K Woo T Klein TA. Off-label prescribing trends for ADHD medications in very young children. J Pediatr Pharmacol Ther. (2017) 22:423–9. doi: 10.5863/1551-6776-22.6.423 PMC5736254 29290742

[23] Greenhill L Kollins S Abikoff H McCracken J Riddle M Swanson J. Efficacy and safety of immediate-release methylphenidate treatment for preschoolers with ADHD. J Am Acad Child Adolesc. Psychiatry. (2006) 45:1284–93. doi: 10.1097/01.chi.0000235077.32661.61 17023867

[24] Childress AC Kollins SH Foehl HC Newcorn JH Mattingly G Kupper RJ. Randomized, double-blind, placebo-controlled, flexible-dose titration study of methylphenidate hydrochloride extended-release capsules (Aptensio XR) in preschool children with attention-deficit/hyperactivity disorder. JCAP. (2020) 30:58–68. doi: 10.1089/cap.2019.0085 32125903 PMC7047252

[25] Childress AC Lloyd E Jacobsen L Gunawardhana L Johnson SA Findling RL. Efficacy and safety of lisdexamfetamine in preschool children with attention-deficit/hyperactivity disorder. J Am Acad Child Adolesc. Psychiatry. (2002) 61:1423–34. doi: 10.1016/j.jaac.2022.03.034 35577034

[26] Sugaya LS Salum GA de Sousa Gurgel W de Morais EM Del Prette G Pilatti CD. Efficacy and safety of methylphenidate and behavioural parent training for children aged 3–5 years with attention-deficit hyperactivity disorder: A randomised, double-blind, placebo-controlled, and sham behavioural parent training-controlled trial. Lancet Child Adolesc. Health. (2022) 6:845–56. doi: 10.1016/S2352-4642(22)00279-6 PMC9731509 36306807

[27] Sugaya LS Farhat LC Califano P Polanczyk GV. Efficacy of stimulants for preschool attention-deficit/hyperactivity disorder: A systematic review and meta-analysis. JCPP Adv. (2023) 3:e12146. doi: 10.1002/jcv2.12146 37720577 PMC10501696

[28] Cortese S Adamo N Del Giovane C Mohr-Jensen C Hayes AJ Carucci S. Comparative efficacy and tolerability of medications for attention-deficit hyperactivity disorder in children, adolescents, and adults: A systematic review and network meta-analysis. Lancet Psychiatry. (2018) 5:727–38. doi: 10.1016/S2215-0366(18)30269-4 PMC6109107 30097390

[29] Wigal T Greenhill L Chuang S McGough J Vitiello B Skrobala A. Safety and tolerability of methylphenidate in preschool children with ADHD. J Am Acad Child Adolesc. Psychiatry. (2006) 45:1294–303. doi: 10.1097/01.chi.0000235082.63156.27 17028508

[30] Houghton R de Vries F Loss G. Psychostimulants/atomoxetine and serious cardiovascular events in children with ADHD or autism spectrum disorder. CNS Drugs. (2020) 34:93–101. doi: 10.1007/s40263-019-00686-4 31768949 PMC6982643

[31] Doyle CA McDougle CJ. Pharmacologic treatments for the behavioral symptoms associated with autism spectrum disorders across the lifespan. Dialogues Clin Neurosci. (2012) 14:263–79. doi: 10.31887/DCNS.2012.14.3/cdoyle PMC3513681 23226952

[32] Harstad E Shults J Barbaresi W Bax A Cacia J Deavenport-Saman A. α2-Adrenergic agonists or stimulants for preschool-age children with attention-deficit/hyperactivity disorder. JAMA. (2021) 325:2067–75. doi: 10.1001/jama.2021.6118 PMC8097628 33946100

[33] Handen BL Johnson CR Lubetsky M. Efficacy of methylphenidate among children with autism and symptoms of attention-deficit hyperactivity disorder. J Autism Dev Disord. (2000) 30:245–55. doi: 10.1023/A:1005548619694 11055460

[34] Research Units on Pediatric Psychopharmacology Autism Network. Randomized, controlled, crossover trial of methylphenidate in pervasive developmental disorders with hyperactivity. Arch Gen Psychiatry. (2005) 62:1266–74. doi: 10.1001/archpsyc.62.11.1266 16275814

[35] Warren AE Hamilton RM Bélanger SA Gray C Gow RM Sanatani S. Cardiac risk assessment before the use of stimulant medications in children and youth: A joint position statement by the Canadian Paediatric Society, the Canadian Cardiovascular Society, and the Canadian Academy of Child and Adolescent Psychiatry. Can J Cardiol. (2009) 25:625–30. doi: 10.1016/S0828-282X(09)70157-6 PMC2776560 19898693

[36] Hirota T Schwartz S Correll CU. Alpha-2 agonists for attention-deficit/hyperactivity disorder in youth: A systematic review and meta-analysis of monotherapy and add-on trials to stimulant therapy. J Am Acad Child Adolesc. Psychiatry. (2014) 53:153–73. doi: 10.1016/j.jaac.2013.11.009 24472251

[37] Mittal S Boan AD Kral MC Lally MD van Bakergem K Macias MM. Young children with attention-deficit/hyperactivity disorder and/or disruptive behavior disorders are more frequently prescribed alpha agonists than stimulants. J Child Adolesc. Psychopharmacol. (2020) 30:81–6. doi: 10.1089/cap.2019.0105 31621385

[38] Deavenport-Saman A Vanderbilt DL Harstad E Shults J Barbaresi W Bax A. Association of coexisting conditions, attention-deficit/hyperactivity disorder medication choice, and likelihood of improvement in preschool-age children: A developmental behavioral pediatrics research network study. J Child Adolesc. Psychopharmacol. (2022) 32:328–36. doi: 10.1089/cap.2022.0009 35787014

[39] Lane SJ Leão MA Spielmann V. Sleep, sensory integration/processing, and autism: A scoping review. Front Psychol. (2022) 13:877527. doi: 10.3389/fpsyg.2022.877527 35656493 PMC9152214

[40] Naguy A. Clonidine use in psychiatry: Panacea or panache. Pharmacology. (2016) 98:87–92. doi: 10.1159/000446441 27161101

[41] Cothros N Martino D McMorris C Stewart D Tehrani A Pringsheim T. Prescriptions for alpha agonists and antipsychotics in children and youth with tic disorders: A pharmacoepidemiologic study. Tremor Other Hyperkinet. Mov. (N Y). (2019) 9. doi: 10.7916/tohm.v0.645 PMC6691607 31413891

[42] Bruni O Angriman M Melegari MG Ferri R. Pharmacotherapeutic management of sleep disorders in children with neurodevelopmental disorders. Expert Opin Pharmacother. (2019) 20:2257–71. doi: 10.1080/14656566.2019.1674283 31638842

[43] Pisano S Masi G. Recommendations for the pharmacological management of irritability and aggression in conduct disorder patients. Expert Opin Pharmacother. (2020) 21:5–7. doi: 10.1080/14656566.2019.1685498 31663786

[44] Galvez-Contreras AY Vargas-de la Cruz I Beltran-Navarro B Gonzalez-Castaneda RE Gonzalez-Perez O. Therapeutic approaches for ADHD by developmental stage and clinical presentation. Int J Environ Res Public Health. (2022) 19:12880. doi: 10.3390/ijerph191912880 36232180 PMC9566361

[45] Eli Lilly and Company. Strattera® (Atomoxetine Hydrochloride) Prescribing Information. Available online at: http://pi.lilly.com/us/strattera-pi.pdf (Accessed 19 March 2024).

[46] Kratochvil CJ Vaughan BS Stoner JA Daughton JM Lubberstedt BD Murray DW. A double-blind, placebo-controlled study of atomoxetine in young children with ADHD. Pediatrics. (2011) 127:e862–8. doi: 10.1542/peds.2010-0825 PMC3387889 21422081

[47] Savill NC Buitelaar JK Anand E Day KA Treuer T Upadhyaya HP. The efficacy of atomoxetine for the treatment of children and adolescents with attention-deficit/hyperactivity disorder: A comprehensive review of over a decade of clinical research. CNS Drugs. (2015) 29:131–51. doi: 10.1007/s40263-014-0224-9 25698145

[48] Alsayouf HA Alsarhan O Khreisat W Daoud A. Atomoxetine treatment of attention deficit/hyperactivity disorder symptoms in 3–6-year-old children with autism spectrum disorder: A retrospective cohort study. Children (Basel). (2024) 11:163. doi: 10.3390/children11020163 38397275 PMC10887200

[49] Ghuman JK Aman MG Ghuman HS Reichenbacher T Gelenberg A Wright R. Prospective, naturalistic, pilot study of open-label atomoxetine treatment in preschool children with attention-deficit/hyperactivity disorder. J Child Adolesc. Psychopharmacol. (2009) 19:155–66. doi: 10.1089/cap.2008.054 PMC2857147 19364293

[50] Davies M Coughtrie A Layton D Shakir SA. Use of atomoxetine and suicidal ideation in children and adolescents: Results of an observational cohort study within general practice in England. Eur Psychiatry. (2017) 39:11–16. doi: 10.1016/j.eurpsy.2016.06.005 27810613

[51] Davies M Coughtrie A Layton D Shakir SA. Use of atomoxetine and suicidal ideation in children and adolescents: Results of an observational cohort study within general practice in England. Eur Psychiatry. (2017) 39:11–6. doi: 10.1016/j.eurpsy.2016.06.005 27810613

[52] Fu D Wu DD Guo HL Hu YH Xia Y Ji X. The mechanism, clinical efficacy, safety, and dosage regimen of atomoxetine for ADHD therapy in children: A narrative review. Front Psychiatry. (2022) 12:780921. doi: 10.3389/fpsyt.2021.780921 35222104 PMC8863678

[53] Fu D Guo HL Hu YH Fang WR Liu QQ Xu J. Personalizing atomoxetine dosing in children with ADHD: What can we learn from current supporting evidence. Eur J Clin Pharmacol. (2023) 79:349–70. doi: 10.1007/s00228-022-03449-1 36645468

[54] Tumuluru RV Corbett-Dick P Aman MG Smith T Arnold LE Pan X. Adverse events of atomoxetine in a double-blind placebo-controlled study in children with autism. J Child Adolesc. Psychopharmacol. (2017) 27:708–14. doi: 10.1089/cap.2016.0187 PMC5651962 28509573

[55] Fung LK Mahajan R Nozzolillo A Bernal P Krasner A Jo B. Pharmacologic treatment of severe irritability and problem behaviors in autism: A systematic review and meta-analysis. Pediatrics. (2016) 137:S124–35. doi: 10.1542/peds.2015-2851K 26908468

[56] Wigal S Chappell P Palumbo D Lubaczewski S Ramaker S Abbas R. Diagnosis and treatment options for preschoolers with attention-deficit/hyperactivity disorder. J Child Adolesc. Psychopharmacol. (2020) 30:104–18. doi: 10.1089/cap.2019.0116 PMC7047251 31967914

[57] Pringsheim T Stewart DG Chan P Tehrani A Patten SB. The pharmacoepidemiology of psychotropic medication use in Canadian children from 2012 to 2016. J Child Adolesc. Psychopharmacol. (2019) 29:740–5. doi: 10.1089/cap.2019.0018 31355670

[58] Brophy S Kennedy J Fernandez-Gutierrez F John A Potter R Linehan C. Characteristics of children prescribed antipsychotics: Analysis of routinely collected data. J Child Adolesc. Psychopharmacol. (2018) 28:180–91. doi: 10.1089/cap.2017.0003 PMC5905863 29486137

[59] Sultan RS Wang S Crystal S Olfson M. Antipsychotic treatment among youths with attention-deficit/hyperactivity disorder. JAMA Network Open. (2019) 2:e197850. doi: 10.1001/jamanetworkopen.2019.7850 31348506 PMC6661708

[60] Lòpez-de Fede A Vyavaharkar M Bellinger JD. Antipsychotic prescriptions for children aged 5 years or younger: Do we need policy oversight standards? SAGE Open. (2014) 4:1–7. doi: 10.1177/2158244014555116

[61] Gleason MM Egger HL Emslie GJ Greenhill LL Kowatch RA Lieberman AF. Psychopharmacological treatment for very young children: Contexts and guidelines. J Am Acad Child Adolesc. Psychiatry. (2007) 46:1532–72. doi: 10.1097/chi.0b013e3181570d9e 18030077

[62] Alsayouf HA Talo H Biddappa ML De Los Reyes E. Risperidone or aripiprazole can resolve autism core signs and symptoms in young children: Case study. Children (Basel). (2021) 8:318. doi: 10.3390/children8050318 33921933 PMC8143447

[63] Alsayouf HA Talo H Biddappa ML. Core signs and symptoms in children with autism spectrum disorder improved after starting risperidone and aripiprazole in combination with standard supportive therapies: A large, single-center, retrospective case series. Brain Sci. (2022) 12:618. doi: 10.3390/brainsci12050618 35625005 PMC9139358

[64] Blader JC Pliszka SR Kafantaris V Foley CA Carlson GA Crowell JA. Stepped treatment for attention-deficit/hyperactivity disorder and aggressive behavior: A randomized, controlled trial of adjunctive risperidone, divalproex sodium, or placebo after stimulant medication optimization. J Am Acad Child Adolesc. Psychiatry. (2021) 60:236–51. doi: 10.1016/j.jaac.2019.12.009 PMC7390668 32007604

[65] Caccia S. Safety and pharmacokinetics of atypical antipsychotics in children and adolescents. Pediatr Drugs. (2013) 15:217–33. doi: 10.1007/s40272-013-0024-6 23588704

[66] Politte LC McDougle CJ. Atypical antipsychotics in the treatment of children and adolescents with pervasive developmental disorders. Psychopharmacology. (2014) 231:1023–36. doi: 10.1007/s00213-013-3068-y 23552907

[67] American Diabetes Association American Psychiatric Association American Association of Clinical Endocrinologists North American Association for the Study of Obesity. Consensus development conference on antipsychotic drugs and obesity and diabetes. Obes Res. (2004) 12:362–8. doi: 10.1038/oby.2004.46 14981231

[68] Ho J Panagiotopoulos C McCrindle B Canadian Alliance for Monitoring Effectiveness and Safety of Antipsychotics in Children (CAMESA) Guideline Group. Management recommendations for metabolic complications associated with second generation antipsychotic use in children and youth. Paediatr Child Health. (2011) 16:575–80. PMC3223901 23115501

[69] Kimura G Kadoyama K Brown JB Nakamura T Miki I Nisiguchi K. Antipsychotics-associated serious adverse events in children: An analysis of the FAERS database. Int J Med Sci. (2015) 12:135–40. doi: 10.7150/ijms.10453 PMC4293178 25589889

[70] Roke Y Van Harten PN Boot AM Buitelaar JK. Antipsychotic medication in children and adolescents: A descriptive review of the effects on prolactin level and associated side effects. J Child Adolesc. Psychopharmacol. (2009) 19:403–14. doi: 10.1089/cap.2008.0120 19702492

[71] Haddad PM Wieck A. Antipsychotic-induced hyperprolactinaemia: Mechanisms, clinical features and management. Drugs. (2004) 64:2291–314. doi: 10.2165/00003495-200464200-00003 15456328

[72] Yilmaz S Serdaroglu G Akcay A Gökben S. Clinical characteristics and outcome of children with electrical status epilepticus during slow wave sleep. J Pediatr Neurosci. (2014) 9:105–9. doi: 10.4103/1817-1745.139266 PMC4166828 25250061

[73] Hergüner MÖ. Incecik F Altunbaşak S Kiriş N. Clinical characteristics of 10 patients with continuous spikes and waves during slow sleep syndrome. Pediatr Neurol. (2008) 38:411–4. doi: 10.1016/j.pediatrneurol.2008.02.007 18486823

[74] Brian JA Zwaigenbaum L Ip A. Standards of diagnostic assessment for autism spectrum disorder. Paediatr Child Health. (2019) 24:444–51. doi: 10.1093/pch/pxz117 PMC6812299 31660042

[75] Nachshen J Garcin N Moxness K Tremblay Y Hutchinson P Lachance A. Screening, assessment, and diagnosis of autism spectrum disorders in young children: Canadian Best Practice Guidelines. Montreal, QC, Canada: Miriam Foundation (2008).

[76] Asakawa T Sugiyama K Nozaki T Sameshima T Kobayashi S Wang L. Current behavioral assessments of movement disorders in children. CNS Neurosci Ther. (2018) 24:863–75. doi: 10.1111/cns.13036 PMC6489758 30039925

[77] Dalsgaard S Østergaard SD Leckman JF Mortensen PB Pedersen MG. Mortality in children, adolescents, and adults with attention deficit hyperactivity disorder: A nationwide cohort study. Lancet. (2015) 385:2190–6. doi: 10.1016/S0140-6736(14)61684-6 25726514

[78] Mahajan R Bernal MP Panzer R Whitaker A Roberts W Handen B. Autism Speaks Autism Treatment Network Psychopharmacology Committee. Clinical practice pathways for evaluation and medication choice for attention-deficit/hyperactivity disorder symptoms in autism spectrum disorders. Pediatrics. (2012) 130:S125–38. doi: 10.1542/peds.2012-0900J 23118243

